# Quality of Source Water and Drinking Water in Urban Areas of Myanmar

**DOI:** 10.1155/2013/854261

**Published:** 2013-06-10

**Authors:** Hiroshi Sakai, Yatsuka Kataoka, Kensuke Fukushi

**Affiliations:** ^1^Department of Urban Engineering, The University of Tokyo, 7-3-1 Hongo, Bunkyo-ku, Tokyo 113-8656, Japan; ^2^Institute for Global Environmental Strategies, 2108-11 Kamiyamaguchi, Hayama, Kanagawa 240-0115, Japan; ^3^Todai Institute for Advanced Study, Integrated Research System for Sustainability Science, The University of Tokyo, 7-3-1 Hongo, Bunkyo-ku, Tokyo 113-8654, Japan

## Abstract

Myanmar is one of the least developed countries in the world, and very little information is available regarding the nation's water quality. This report gives an overview of the current situation in the country, presenting the results of various water-quality assessments in urban areas of Myanmar. River, dam, lake, and well water sources were examined and found to be of generally good quality. Both As and F^−^ were present in relatively high concentrations and must be removed before deep wells are used. Heterotrophic plate counts in drinking water were highest in public pots, followed by nonpiped tap water, piped tap water, and bottled water. Measures need to be taken to improve low-quality water in pots and nonpiped tap waters.

## 1. Introduction

Access to safe water is a significant issue in developing countries. According to a WHO report, around 780 million people globally do not have access to adequate water supply sources [1]. Additionally, 2.5 billion people do not have access to suitable sanitation facilities. Furthermore, about 2 million people die every year due to diarrheal diseases. Therefore, access to safe water is a crucial requirement in developing countries, where infrastructure is not always provided and often needs to be expanded. Due to the mismatches of urban planning and actual residential area, some areas must rely on inadequate private water supplies. This is a distinct issue in slum area and periurban areas.

Myanmar is a developing country in Southeast Asia. Even among the developing countries, Myanmar falls into the category of least developed countries by United Nations criteria [2]. The water infrastructure needs to be developed for the country's further economic development. However, very few water-quality data are currently available. To the best of our knowledge, only a single water-quality survey has been conducted [3]. That study reported water quality in Lake Inle in the northeastern part of the country. Some water-quality data for dams are provided on the webpage of the Water Environment Partnership in Asia [4] but only for limited quality parameters. To our knowledge, no other information has been reported, and the current water-quality and sanitation situations in Myanmar therefore remain unclear. We visited the country to perform a water-quality survey and assess the current situation with respect to water infrastructure. The survey was conducted in two urban areas, Yangon and Nay Pyi Taw, the former and current capital, respectively. This article reveals the water-quality and sanitation situations in Myanmar for the first time.

## 2. Materials and Methods

### 2.1. Study Area

**Figure 1:**
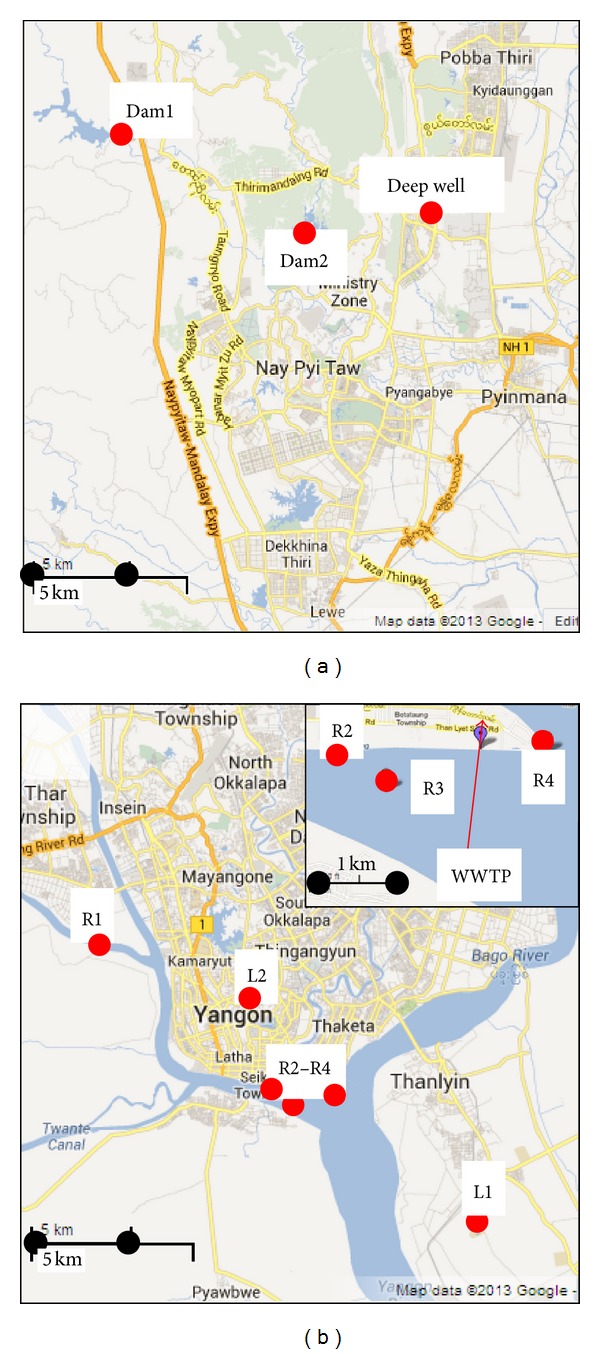
Maps of sampling locations in (a) Nay Pyi Taw and (b) Yangon.

 We surveyed two urban areas, Nay Pyi Taw and Yangon. The city of Nay Pyi Taw became the capital in 2008; the city of Yangon was the previous capital. A survey of drinking-water sources and quality was undertaken. The details of the location of source waters are shown in Figure 1. In Nay Pyi Taw, source waters from a deep well and two dams were examined. In the Yangon area, environmental waters in lakes and rivers were examined. 

 Drinking water was collected from various sources including public pots, nonpiped taps, piped taps, and bottled waters, as shown in Table 1. Three bottled waters (500 mL) from different companies were obtained commercially. Piped tap water was collected from three taps in Yangon and Nay Pyi Taw. Nonpiped tap water was collected at a pagoda and at another building (building D). In Myanmar, a pagoda is a meeting place for Buddhists, and complementary drinking water is provided. The tap water at the pagoda was provided by a nonpiped supply source. It was treated by a point-of-use (POU) facility and then stored. Building D was situated outside Yangon city in an area that is not served by the Yangon City Development Committee (YCDC) tap-water service. The water supply system to the building was privately operated by a POU facility that used a combination of a reverse osmosis (RO) membrane treatment and an ultraviolet (UV) disinfection system.

**Figure 2:**
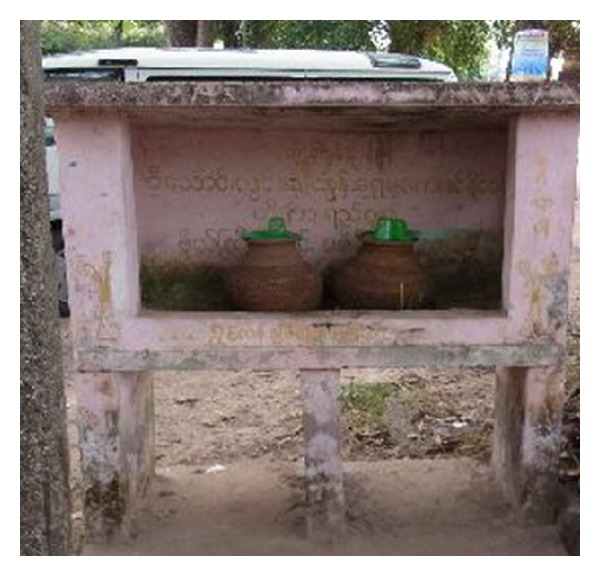
Roadside pots containing water for drinking.

 Drinking water was also collected from public pots located on the roadside (Figure 2). In the Yangon area, pots are filled with water, covered, and placed along the roadside for public drinking purposes. We collected samples from various pots and examined the water quality, with a focus on bacterial analysis. 

### 2.2. Measured Parameters

#### 2.2.1. Bacteria

The numbers of *E. coli* and total coliform bacteria and the heterotrophic plate counts (HPC) were determined using a commercial kit (Petrifilm, 3M, USA) at each site. *E. coli* and total coliform bacteria were incubated at 37°C for 24 hours, and HPC was incubated at 37°C for 48 hours. The validity of this kit has been confirmed [5, 6], and it showed high correlations for *E. coli* and total coliform. High correlations were also reported for HPC, although small differences can arise with different incubation conditions [7]. 

#### 2.2.2. Chemical Parameters

Dissolved organic carbon (DOC), dissolved total nitrogen (DTN), and anions were measured with a TOC analyzer (TOC-L, Shimadzu). Anion concentrations (F^−^, Cl^−^, Br^−^, NO_2_^−^, NO_3_^−^, PO_4_^−^, and SO_4_^−^) were determined by ion chromatography (IC-861, Metrohm) after filtration through a 0.45 *μ*m polytetrafluoroethylene (PTFE) membrane. 

#### 2.2.3. Heavy Metals

Dissolved metals were analyzed by ICP-MS (7500 Series, Agilent) after filtration through a 0.45 *μ*m PTFE membrane.

## 3. Results and Discussions

### 3.1. Source Water Quality in Nay Pyi Taw

**Figure 3:**
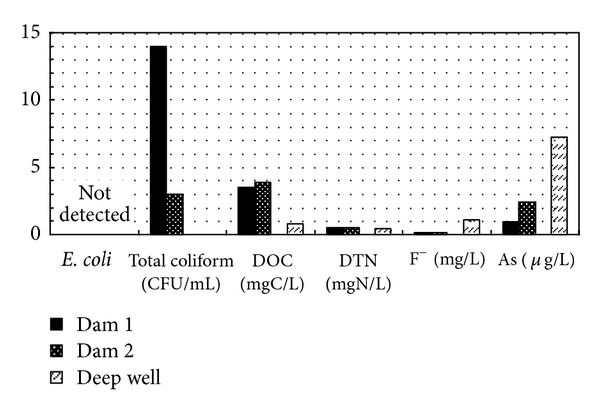
Comparison of water quality in dams and a deep well water at Nay Pyi Taw.

Water quality parameters in two dams and a deep well in Nay Pyi Taw were examined and compared. A summary of the results, shown in Figure 3, indicates generally good water quality. No *E. coli* was detected in 1 mL samples at all locations, indicating good bacterial water quality. Total coliform levels were 14 and 3 CFU/mL at the two dams. These values are close to 10 CFU/mL, which is the “class A" Japanese environmental standard for lake water [8]. The DOC was 3.5 mg/L at Dam 1 and 3.0 mg/L at Dam 2, and it was 0.8 mg/L in the deep well. Values of DOC at the two dams were acceptable for water sources, considering that 3 mg/L of TOC in finished water has been adopted as a drinking-water standard in Japan [9]. A previous study had reported about 30–50 mg C/L of TOC in Lake Inle [3]. Compared with the results for Lake Inle, these two dams have a much better water quality. 

 Two important observations were made with regard to the deep well water quality. Fluoride ion levels were 1 mg/L, which is close to the 1.5 mg/L WHO guideline value [10]. The As level was 7.9 *μ*g/L, which is also close to the WHO guideline value of 10 *μ*g/L. Therefore, adequate water treatment must be provided before the well can be used as a drinking-water source. Overall, the water quality in the two dams and the deep well could be considered fair, and the water could be used for drinking with appropriate treatment. 

### 3.2. Environmental Water Quality in Yangon

Environmental water quality was also surveyed in the Yangon region. The results of bacterial parameters are shown in Table 2. The Japanese standard for total coliform levels in river water is 0.5 CFU/mL for class AA, 10 CFU/mL for class A, and 50 CFU/mL for class B [8]. River waters in Yangon were found to be close to the class B standard, indicating that they can be used for drinking after advanced treatment. Among the sampling points on the Yangon River, R2 was located on the left riverbank, whereas R3 and R4 were located in the center of the river. The total coliform value was highest at R2 on the left riverbank, which was closest to urban activity. There was also a discharge from a wastewater treatment plant on the left riverbank, which would have contributed to the larger number of total coliform recorded at R2. 

L1 and L2 are recreational lakes in the Yangon region. At L1, *E. coli* was not detected in 1 mL samples, whereas the level was 2 CFU/mL at L2. Total coliform was also high, with 110 CFU/mL at L2. A previous report indicated total coliform levels of 18–137 CFU/mL in Lake Inle [3]. Considering these numbers, L2 was not suitable as a drinking-water source.

**Figure 4:**
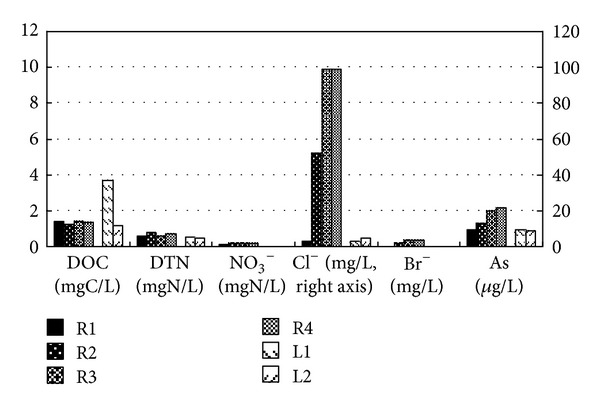
Comparison of water quality in rivers and lakes at Yangon.

The chemical water-quality parameters are summarized in Figure 4. In river water samples, the DOC was less than 3 mg C/L, which satisfies the Japanese drinking-water-quality standard [9]. Levels of the Cl^−^ ion tended to increase downstream. The levels of Br ion and As displayed a similar trend, although the decrease was not as marked. In contrast to the elemental measurements, DOC, DTN, and nitrate were stable along the river flow. It was therefore assumed that the sources of Cl^−^, Br^−^, and As were different from those of carbon and nitrogen.

Samples from L1 and L2 had good water quality with regard to chemical parameters, except for high DOC in L1. Overall, lake and river waters were good in terms of their chemical parameters, but the levels of bacterial contamination needed improvement.

### 3.3. Drinking-Water Quality

Potable drinking-water quality was surveyed for various water sources in Myanmar including (i) public pots, (ii) piped water supply in Yangon and Nay Pyi Taw, (iii) nonpiped water supply, and (iv) bottled water. 

**Figure 5:**
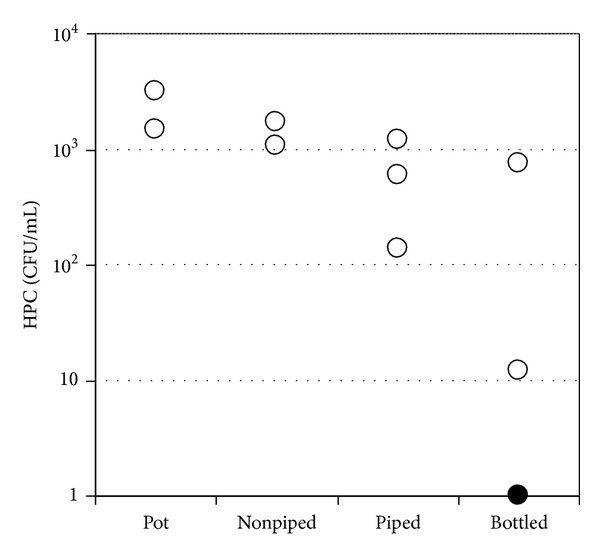
Heterotrophic plate count for potable water.

For bacterial water quality, *E. coli* was not detected in 1 mL water samples from any water source. However, there was a clear trend in the HPC, as shown in Figure 5. Pot water had HPC levels of 3200 and 1500 CFU/mL. In nonpiped tap water, the HPC level was 1700 and 1170 CFU/mL, and, in piped tap waters, the HPC was 140, 600, and 1200 CFU/mL. Of the three bottled waters examined, HPC was detected in two bottles at 760 and 12 CFU/mL, but HPC was not detected in the other bottled water sample. HPCs have various incubation conditions [11], and different incubation conditions produce different values. The Japanese government has adopted a value of 2000 CFU/mL as a water-quality standard for HPC by incubation at 20°C for 7 days [9]. This is equal to about 740 CFU/mL by incubation at 37°C for 2 days in Petrifilm, which we used in our preliminary investigation (unpublished data). From these results, we concluded that water from all pots, all nonpiped taps, one piped tap, and one bottled water exceeded the converted value of the Japanese drinking-water-quality standard and may not be suitable for drinking. 

As shown in Figure 5, HPC was highest in the pots, followed by nonpiped taps, piped taps, and bottled waters. Nonpiped water was surveyed at two taps, at a pagoda and at a building outside the YCDC tap water service area. At those taps, water was supplied after treatment by POU facilities. 

Piped water was surveyed at two buildings in Nay Pyi Taw and at one building in Yangon. Piped water supplies contained some residual chlorine: 0.01 mg/L at two taps in Nay Pyi Taw and 0.03 mg/L at a tap in Yangon. At a tap in Yangon, chlorine was present entirely as free chlorine. This residual chlorine may have contributed to the upkeep of water quality in the piped water supply. 

It is interesting to note the water-quality distribution in bottled waters, with one bottled water supply having worse water quality than two piped taps. This probably results from the source water quality and the treatment efficiency of different bottled water companies. Other water-quality parameters were also monitored, including As and F^−^. All measured items satisfied the Japanese drinking-water-quality standards [9].

### 3.4. Treatment Efficiency of a POU Facility

**Figure 6:**
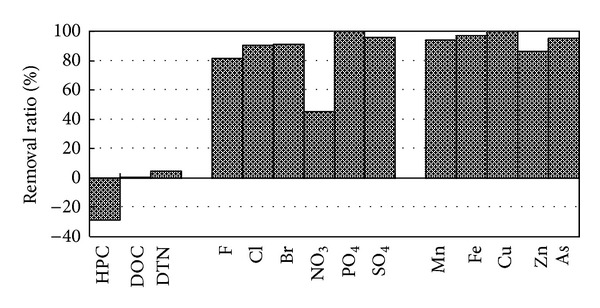
Removal ratio for a point-of-use (POU) facility.

We investigated the performance of a POU facility at a building in Yangon, which was situated outside the YCDC piped water supply area. The water source was ground water, which was treated by an RO membrane followed by UV disinfection. The removal ratios of bacteria (HPC), carbon and nitrogen, anions, and metals are shown in Figure 6. The listed heavy metals were removed with high efficiency. Anions were also removed with high efficiency except for nitrate, for which only a 45% removal was achieved. Because UV treatment does not remove anions and metals, these elements were removed by the RO membrane. 

In contrast, DOC and DTN removal was very low: 1% DOC and 5% DTN. The DOC and DTN contents of raw water were 0.55 mg C/L and 0.23 mg N/L, respectively. Considering the removal of anions and metals, the RO membrane would have worked well. A possible explanation is that most organic matter and nitrogen in raw water have a very small molecular weight and can pass through the RO membrane. Further investigation of the molecular weight distribution would confirm this assumption. 

It is noteworthy that the bacterial removal ratio was negative. Considering the removal of anions and metals, bacteria could be removed by the RO membrane treatment. UV treatment also contributes to the suppression of bacterial activity, but UV treatment has no residual effect. Therefore, bacterial regrowth may occur in a storage tank after UV treatment. UV treatment has a high potential to be installed in POU facilities because of its ease of handling and maintenance. Maintaining bacterial water quality after UV treatment is an important issue outside the piped water supply area.

## 4. Conclusion

This study investigated the water quality in urban areas of Myanmar and produced an overview of the current situation. River, dam, lake, and well water samples were examined and found to be of generally good quality. As and F^−^ were present at relatively high concentrations and must be removed before deep wells can be used. Heterotrophic plate counts in drinking water were highest in pots, followed by nonpiped tap water, piped tap water, and bottled water samples. Measures need to be taken to improve the poor water quality in pots and nonpiped taps.

## Figures and Tables

**Table 1:** Summary of drinking-water samples.

Type	Description	Location
Pot	Pot A	Yangon
	Pot B	Suburban Yangon

Nonpiped	Pagoda A	Yangon
	Building D	Suburban Yangon
	After treatment	
	Before treatment	

Piped	Building A	Yangon
	Building B	Nay Pyi Taw
	Building C	Nay Pyi Taw

Bottle	Manufacturer A	—
	Manufacturer B	—
	Manufacturer C	—

**Table 2:** Bacterial water quality in Yangon.

Location	R1	R2	R3	R4	L1	L2
Total coliform (CFU/mL)	52	820	32	38	10	110
*E. coli *(CFU/mL)	20	510	15	16	<1	2
